# The combination of green tea, caffeine, conjugated linoleic acid and branched chain amino acids have no effect on body composition and abdominal fat changes in overweight and obese men and women

**DOI:** 10.1186/1550-2783-9-S1-P29

**Published:** 2012-11-19

**Authors:** D David Thomas, Shweta Rawal, Amber W Kinsey, Wyatt E Eddy, Nicholas Fisher, Maria M Spicer, Michael J Ormsbee

**Affiliations:** 1 Department of Nutrition, Food and Exercise Science, The Florida State University, Tallahassee, FL 32306, USA

## Background

Caffeine, conjugated linoleic acid (CLA), green tea and branched chain amino acids (BCAA) have shown to individually improve body composition in overweight and obese men and women. The purpose of this study was to investigate the effects of a multi-ingredient dietary supplement containing caffeine, CLA, green tea, and BCAA on body composition and abdominal fat mass in overweight and obese men and women.

## Methods

Thirty-four healthy men and women were randomly assigned to two groups: 1) a soybean oil placebo (PL) or 2) a multi-ingredient dietary supplement (DS) containing 99 mg of caffeine and a propriety blend containing 1510 mg of CLA, green tea extract (45% EGCG), L-leucine, L-isoleucine and L-valine. Twenty-two participants completed the study (PL: n=11; age, 34 +12 years; body mass, 97.0 + 22.6 kg; BMI, 34.1 ± 6.1; DS n=11; age, 36+ 11.1 years; body mass, 91.9 + 18.7 kg; BMI, 30.0 + 4.9). Both groups consumed two pills with breakfast and two pills with lunch. Body composition and android fat (dual-energy X-ray absorptiometry), waist and hip circumferences, blood pressure and heart rate were measured at baseline and after 8 weeks of supplementation. Participants were instructed to maintain normal dietary and exercise habits for the duration of the study. Data was analyzed using JMP 9 Pro (Cary, NC), significance was set to p<0.05. A two-way ANOVA with repeated measurements was used to evaluate changes in dependent variables over time ([Pre x Post] x [PL x DS]). If significant time, group, or group-by-time interactions were reported, a Tukey test was used for post hoc comparisons.

## Results

Twenty two participants finished the study. Five participants dropped the study due to personal reasons and seven were excluded from the data due to low compliance (<80%) to the supplement. No significant changes were measured in body composition, android fat, waist or hip circumference, heart rate and blood pressure.

## Conclusion

Eight weeks of supplementation of a multi-ingredient supplement containing caffeine, CLA, green tea, and BCAA did not affect body composition, android fat, heart rate, or blood pressure in overweight and obese men and women.

